# Using Embedded Feature Selection and CNN for Classification on CCD-INID-V1—A New IoT Dataset

**DOI:** 10.3390/s21144834

**Published:** 2021-07-15

**Authors:** Zhipeng Liu, Niraj Thapa, Addison Shaver, Kaushik Roy, Madhuri Siddula, Xiaohong Yuan, Anna Yu

**Affiliations:** Computer Science Department, North Carolina Agricultural and Technical State University, 1601 E Market St, Greensboro, NC 27411, USA; zliu2@aggies.ncat.edu (Z.L.); nthapa@aggies.ncat.edu (N.T.); awshaver@aggies.ncat.edu (A.S.); msiddula@ncat.edu (M.S.); xhyuan@ncat.edu (X.Y.); cshmyu@ncat.edu (A.Y.)

**Keywords:** IDS, IoT, deep learning, dataset, feature selection, dimension reduction, random forest, XGBoost, feature engineering, hybrid models

## Abstract

As Internet of Things (IoT) networks expand globally with an annual increase of active devices, providing better safeguards to threats is becoming more prominent. An intrusion detection system (IDS) is the most viable solution that mitigates the threats of cyberattacks. Given the many constraints of the ever-changing network environment of IoT devices, an effective yet lightweight IDS is required to detect cyber anomalies and categorize various cyberattacks. Additionally, most publicly available datasets used for research do not reflect the recent network behaviors, nor are they made from IoT networks. To address these issues, in this paper, we have the following contributions: (1) we create a dataset from IoT networks, namely, the Center for Cyber Defense (CCD) IoT Network Intrusion Dataset V1 (CCD-INID-V1); (2) we propose a hybrid lightweight form of IDS—an embedded model (EM) for feature selection and a convolutional neural network (CNN) for attack detection and classification. The proposed method has two models: (a) RCNN: Random Forest (RF) is combined with CNN and (b) XCNN: eXtreme Gradient Boosting (XGBoost) is combined with CNN. RF and XGBoost are the embedded models to reduce less impactful features. (3) We attempt anomaly (binary) classifications and attack-based (multiclass) classifications on CCD-INID-V1 and two other IoT datasets, the detection_of_IoT_botnet_attacks_N_BaIoT dataset (Balot) and the CIRA-CIC-DoHBrw-2020 dataset (DoH20), to explore the effectiveness of these learning-based security models. Using RCNN, we achieved an Area under the Receiver Characteristic Operator (ROC) Curve (AUC) score of 0.956 with a runtime of 32.28 s on CCD-INID-V1, 0.999 with a runtime of 71.46 s on Balot, and 0.986 with a runtime of 35.45 s on DoH20. Using XCNN, we achieved an AUC score of 0.998 with a runtime of 51.38 s for CCD-INID-V1, 0.999 with a runtime of 72.12 s for Balot, and 0.999 with a runtime of 72.91 s for DoH20. Compared to KNN, XCNN required 86.98% less computational time, and RCNN required 91.74% less computational time to achieve equal or better accurate anomaly detections. We find XCNN and RCNN are consistently efficient and handle scalability well; in particular, 1000 times faster than KNN when dealing with a relatively larger dataset-Balot. Finally, we highlight RCNN and XCNN’s ability to accurately detect anomalies with a significant reduction in computational time. This advantage grants flexibility for the IDS placement strategy. Our IDS can be placed at a central server as well as resource-constrained edge devices. Our lightweight IDS requires low train time and hence decreases reaction time to zero-day attacks.

## 1. Introduction

Not only has the number of smart devices connected significantly grown, the world has also witnessed a sharp increase in IoT applications in numerous smart environments [1]. Echoing this growth is the escalating number of cyberattacks [2,3,4]. Developing countermeasures to safeguard the security of these networks and the privacy of users cannot be taken lightly [5,6]. The top choice of these countermeasures is an IDS [7,8].

However, with the complexity of IoT network topology and the diversification of intrusion behavior, the existing intrusion detection technologies have presented some drawbacks: 

(1) Dynamic and scalable environment—The first challenge is the vast variations in the applications of IoT systems used in recent years [9,10,11]. The areas include home, campus, transportation, manufacture, retail, and smart city infrastructures, with rapid developments in wireless communication, smartphone, healthcare, smart grid, home automation, distributed pollution monitoring, smart lighting systems, and sensor network technologies. Depending on the IoT scenario, the number of connected devices can range from several to millions. These applications and the number of connected devices contribute to a larger attack surface, which means higher difficulty detecting and mitigating the attacks. 

(2) Big data to limited resource—Increased devices generate overhead traffic that essentially becomes big data, which means higher-dimensional data. Since most IoT devices have low computing power, the storage or capture of this data becomes challenging as well. A malicious entity could generate a flood of messages to consume the limited resource on IoT edge devices and create a denial of service (DoS) to legitimate users [12] or even hold ransom for their rightful information [13].

(3) Shortages of public datasets—Another notable challenge is the unavailability of publicly available training datasets [14]. Effective utilization of machine learning (ML) and deep learning (DL) needs recently developed datasets that carry the latest cyberattacks [15].

Most of the current research has explored different publicly available datasets, including DARPA/ KDD99 [16], created in 1999. An extended version of KDD, namely NSL-KDD [17], is available with new features. However, these datasets do not reflect the modern-day networks filled with IoT devices. While widely accepted as benchmarks, these datasets no longer represent contemporary zero-day attacks and the IoT ecosystem [18]. In [19], a new real-time packet-based dataset named IoT-DDoS is collected using multiple protocols. However, the dataset only carries 12 features, far less than the 41 features in KDDcup99. In [20], a holistic smart home framework combined with a multi-facet dataset is introduced. But evaluation metrics are lacking from this work. In [21], researchers from Stratosphere Laboratory produced a dataset to solely facilitate the detection of IoT-based botnets. Even though there are some alternatives available, it is not close to enough [22].

(4) Limited and non-standardized features—Feature engineering for IoT data is another challenge. The detection rate of an IDS is heavily dependent on the features used in training. The performance of IDS often varies when different sets of features of network data are used. Publicly available datasets do not have a standardized set of features [22].

Plenty of research work in recent years has been dedicated to secure IoT networks [23]. Detecting anomalies from benign traffic or identifying various attacks from typical network datasets using only traditional ML approaches have seen great success [24,25,26]. However, traditional ML approaches struggle when dealing with a large volume of IoT data [22,27,28].

DL is offered as a solution to overcome the shortcomings of ML approaches when dealing with big data [29]. DL applies an artificial neural network (ANN) to analyze improbable data for human minds to comprehend [30,31]. Given the amount of data at the volatile speed of transmissions, DL is a definite choice for classifying attacks in IoT networks. Unfortunately, DL has disadvantages as well. DL requires a large amount of data with a sizeable number of features, which means data is usually high dimensional. However, given the limited amount of computational power on edge devices, retraining the models with new inputs proves challenging. DL does not perform well with limited data, even with an optimized parameter set [22]. Optimally, lightweight IDS [32], which is a powerful yet small and flexible form of IDS, is a preferable choice for edge and fog networks. However, most DL-based IDSs are not lightweight. Another setback is that even though DL can perform the feature selection, the selected features may lack context and remain a black box myth that is hard to explain [33,34]. Alternatives to feature selection using DL is the embedded methods. Given a dataset, the main objective of feature selection is to identify the best set of features that generate the most optimal results from models. Taxonomically, feature selection methods are classified as: Filter, Wrapper, and Embedded [35]. By analyzing univariate stats, Filter methods select the inherent characteristics from features. Examples of Filter methods include linear discriminant analysis (LDA), analysis of variance (ANOVA), and chi-square. Compared to wrapper methods, which search for the spatial relationships between all feature subsets using a greedy algorithm, the computational requirement is less. However, wrapper methods often produce higher predictive results than filter methods [35]. Some wrapper methods include forward selection, backward elimination, and recursive feature elimination. Embedded methods encompass the benefits of both the wrapper and filter methods, by including interactions of features but also maintaining reasonable computational cost. Embedded methods, such as decision trees, RF, and XGBoost, iteratively pick the most meaningful features for training in that iteration. Recently, RF and XGBoost have been shown promising performances in selecting the most important features [36,37,38]. Given a dataset with reduced features, producing a model that does not compromise detection rate while utilizing the advantages of DL is significant. However, if the wrong features are selected, the model can be flawed and underperform [39,40]. Finding the correct combination of feature selection and the predictive model is the key.

Based on these facts, an efficient IDS method: (1) should be lightweight and handle both the limited and large amount of data without demanding too much computational power [41], (2) can detect zero-day and complex attacks, and (3) can extract useful features [42].

In this research, we created a publicly available dataset using smart sensors in an IoT network and propose a new lightweight IDS based on a hybrid model.

Our contributions are three-fold:

- To demonstrate a real-world attack scenario and evaluate the effectiveness of our proposed IDS, we create an IoT network-based dataset, namely, Center for Cyber Defense (CCD) IoT Network Intrusion Dataset V1 (CCD-INID-V1). The data is collected in the smart lab and smart home environments using Rainbow HAT sensor boards installed on Raspberry Pis.
- To provide a solution to devise resource constraints and utilize IDS placement, we propose a lightweight and hybrid technique for IoT intrusion detections. The placement of IDS for IoT networks are primarily in: cloud [43,44], fog [45], and edge [46]. In this work, we adopt a hybrid format [47], which is a combination of fog computing and cloud computing. We monitor and generate features at the fog layer and compute detection training and testing at the cloud layer. Our proposed hybrid method combines an embedded model (EM) for feature selection and a CNN for attack classification. The proposed intrusion detection method has two models: (a) RCNN, where RF is combined with CNN, and (b) XCNN, where XGBoost is combined with CNN. The EM selects the most influential features without compromising the detection rates.
- To compare the effectiveness of our proposed technique to traditional ML algorithms, such as k-nearest neighbors (KNN), naïve bayes (NB), logistic regression (LR), and support vector machine (SVM), we use two publicly available datasets, the detection_of_IoT_botnet_attacks_N_BaIoT dataset (Balot) [48], and the CIRA-CIC-DoHBrw-2020 dataset (DoH20) [49], as benchmarks and provide the comparative results of anomaly and multiclass classifications.

The rest of this paper is organized as follows. We briefly introduce the related research work in Section 2, especially feature selections with traditional models and classifications using DL techniques in intrusion detection. In Section 3, we discuss the proposed methodologies and introduce the three datasets. Section 4 describes the design and implementation in details. Section 5 shows the experimental results. Section 6 concludes the paper and provides future research directions.

## 2. Related Work

Most IDSs classify attacks by analyzing network traffic generated from specialized environments [50,51,52,53,54,55]. Nevertheless, in reality, network traffic may originate from a broad range of traffic and include excessive data. A sound IDS should be able to extract meaningful data and correctly classify malicious traffic from benign traffic. This section discusses the related work in the context of feature reduction and DL-based anomaly and intrusion detection.

The embedded feature selection scheme has been preferred over the filter and wrapper methods [56,57,58], and has seen success in fields such as bioinformatics [59,60], and medical research [61,62,63,64], but remains relatively new in the field of IoT security.

Although many have used feature selection algorithms such as Principal Component Analysis (PCA) [65,66], KNN [67,68], NB [69,70], LR [71,72], but recent works predominantly use RF [73,74,75,76,77] and XGBoost [78,79,80,81,82]. In particular, the authors in [83] provide a detailed analysis of RF-based feature selection. They were able to select the meaningful features and reduce the dimension from 41 to 25 based on a score metric. The RF-based model maximized the rate of performance and minimized the false positive rate for IDS. In [84], the authors proposed an anomaly-based IDS using traditional ML algorithms, in particular SVM. The traditional ML-based scheme reported in [84] applies a fitness function to reduce the feature dimension, increase true positive rate and simultaneously, decrease the false positive rate. In [23], to compare the effectiveness of feature reduction, RF is compared with PCA, NB and several filter methods. RF performed the best out of the compared methods without significantly compromising model efficiency.

Jashuva et al. [85] stressed the importance of attribute or feature selection for performing accurate network intrusion detection through manual feature selection. They increased accuracies by only selecting the top 20 features with a cutoff threshold value. However, manually selecting features is time consuming and labor intensive.

In [86,87], the authors proposed to use auto encoders to extract features from datasets and reduce feature dimensions. The proposed approach results in reduced memory usage and improved attack detections. However, the auto encoders were not used for anomaly detection.

Sakurada et al. [88] proposed the utilization of a self-encoder in anomaly detection. The auto encoder is applied to artificial and real data to reduce dimensions. The performance was compared with linear and kernel PCA. But the method was not lightweight and it was not applied to network intrusion detection. Here, we note that an appropriate feature extraction framework is very helpful to speed up computational efficiency.

In [89], to reduce the feature size, a method called Jumping Gene adapted NSGA-II multi-objective optimization was applied. CNN integrated long short-term memory (LSTM) was used to classify the distributed denial-of-service attack (DDoS). However, the work only examined a single attack from a single dataset, the CISIDS2017 dataset [85].

Zhong et al. [90] compared the results from two new DL methods, Gated Returning Units (GRU) and Text-CNN, with traditional ML algorithms such as Decision Tree, NB and SVM. The methods were applied on two datasets: KDD99 [17] and the ADFA-LD [91]. GPU is set up to have two gates: rest gate r and update gate z. The reset gate is used to merge new input with previous stored information, and the update gate manages the amount of previous stored information on the current time step. Text-CNN is a neural network built from trained word vectors. Text-CNN is applied as an embedding layer. Both methods were designed as language models but were used to sequential analyze tcpdump packets to collect features. The paper concluded that the two new DL methods outperform other methods in terms of F-1 score.

Shurman et al. [92] proposed two models in an attempt to detect anomalies on the CICDDoS2019 dataset [93]. The first model was a hybrid model that encompasses signature-based method with an anomaly-based method. The second model is an LSTM model. However, the work only attempted to detect a specific DoS attack and the methods were not applied on various datasets.

To the best of our knowledge, we are the first to combine the EM-based feature selectors with deep neural networks (DNNs) in the field of IDS in an IoT setting. Table 1 shows a comparison of different IDS schemes.

## 3. Methods and Datasets

This section describes the architectures for the proposed models and introduces the three datasets used to assess the models.

Both the proposed models, RCNN and XCNN, utilize EM to select the meaningful features to reduce feature dimensions. The data with reduced dimension is then fed into the DL-based CNN. The models were applied for binary classification to detect cyber anomalies and multiclass classification to classify various types of cyber-attacks. Our CCD-INID-V1 dataset contains five types of cyberattacks. The Balot contains ten types of cyberattacks [48], and DoH20 contains three types of cyberattacks [49]. Each dataset used in this research represents a non-overlapping and distinct set of attacks to show the effectiveness of the proposed models. For comparative analysis, we apply the RCNN and XCNN models on three datasets and compare the performances with the traditional ML models.

### 3.1. Architectures for RCNN and XCNN

In this section, we will discuss the proposed RCNN and XCNN models. While RCNN uses RF to select meaningful features, XCNN uses XGBoost.

The process begins when we train the pre-processed data using the EM-based feature selectors. Feature selection, either manual or automatic, is used to select the most desired features contributing to the predictive outcomes. The necessity of such an act can be sourced to the curse of dimensionality. This refers to a group of phenomena where the data has many dimensions but is sparse. By reducing the number of features to process, fewer dimensions need to be examined by the models, making the data less sparse and statistically significant for ML applications. Feature reduction through feature selection leads to the need for fewer resources to complete the computations or tasks. Feature reduction removes multicollinearity resulting in improvement of the ML model in use. The irrelevant or less meaningful features for training may decrease the prediction accuracy of the model and take huge computational effort. The benefit of selecting the most optimized feature selector is a crucial component of an effective IDS. To minimize the IDS run time and inaccurate detection rate, and develop a lightweight and accurate IDS scheme, we applied RF as a feature selector for the RCNN model and XGBoost for the XCNN model. Using the CCD-INID-V1 dataset, we were able to reduce the input from an original set of 83 features to an optimal subset of 41 features. Data input is significantly reduced, and the most relevant features were retained. The remaining features were used to train the model and validate the test data.

As mentioned, our RCNN model uses the RF algorithm to select impactful features. The RF model is an ensemble tree-based learning algorithm and is a well-known feature selection technique. RF generates possible trees against the target attribute to elicit the important features. Statistical usage of different attributes is calculated, and using the same, the most informative subset of features is found. If an attribute is often selected as best split, then it is retained. A tree-based model involves recursively partitioning the given dataset into two groups based on a certain criterion until a predetermined stopping condition is met. In a tree, we calculate how many times an attribute is selected as best split and based on it the attribute is ranked. Attributes with higher rank are considered in the dimensional space. Unlike decisions tress, which are prone to overfitting, RF utilizes the technique of bootstrap aggregating to reduce the possibility of overfitting [96].

XCNN optimizes the selection of features with the help of XGBoost. XGBoost is a library of gradient boosting algorithms optimized for modern data science problems and tools [97]. First, XGBoost is one of the most popular boosting tree algorithms for gradient boosting machine (GBM). It leverages the techniques mentioned with boosting. Some of the major benefits of XGBoost are that it is highly scalable/parallelizable, quick to execute, and typically outperforms other algorithms [98,99].

After feature selection, the reduced data is fed into CNN. Our CNN model has the following configurations:

- An embedding layer of batch size 512
- A convolutional 2D layer of size 64 × 64 using RELU activation function
- A dropout layer with rate of 0.3
- A convolutional 2D layer of size 128 × 128 using RELU activation function
- A maxpooling layer
- A flatten layer
- A dense layer of size 128
- A dense layer of size 64
- A dropout layer with rate of 0.3
- A dense layer of size 16
- An output layer of 2 or n classes using Adam optimizer

**Figure 1:**
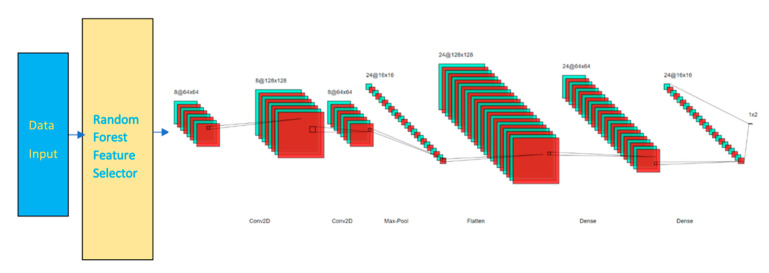
RCNN Model.

**Figure 2:**
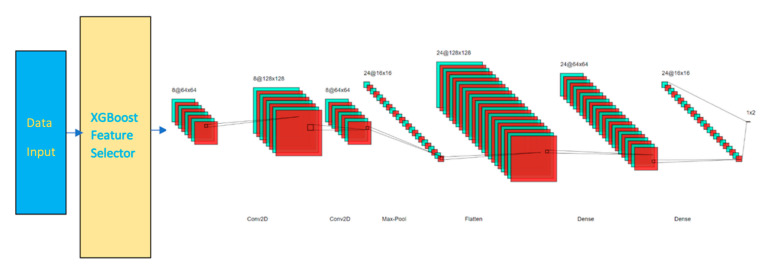
XCNN Model.

As shown in Figure 1 and Figure 2, the CNN is built in a sequential order. The embedding layer enables us to convert each feature input into a fixed length vector of defined size. The resultant vector contains real numbers instead of 0′s and 1′s. The vector represents data relationships in another perspective without increasing the dimension at relatively low computation cost. We selected 512 as our batch size.

Two convolutional layers with respective sizes of 64 multiplied by 64 and 128 multiplied by 128 were added with the activation function of rectified linear unit (RELU). RELU is a linear function that outputs the input directly if is positive, or else it will output zero. A dropout layer of 30% dropout rate is added to avoid overfitting. A maxpooling layer is then included to progressively reduce the spatial size of the representation. The layer reduces the computational cost by reducing the number of parameters to learn and provides basic translation invariance to the internal representation, otherwise known as the sample-based discretization process.

The flatten layer reshapes the values from the previous layer into one-dimensional before the values pass through two dense layers. Dense layers look the values in non-linear views. Another dropout layer with 30% dropout rate is added before another dense layer. In the final layer, adaptive moment estimation (Adam) optimizer is used to tune the parameter values. The parameter of number classes is set to either 2 or n depending on the expected outcomes is binary or multiclass in nature. The model is trained over 10 epochs.

### 3.2. Datasets Used

The following section discusses the three datasets used for evaluating our models in detail.

#### 3.2.1. CCD IoT Network Intrusion Dataset V1

We collected and developed the CCD-INID-V1 dataset at Center for Cyber Defense, North Carolina A&T State University.

This section discusses the data collection process. In [100], Ullah et al. compare the setup to various datasets. The compared datasets simulate traffic to mimic real-world networks. The data generations originate from both physical and virtual devices. Most of these datasets are created in virtual environment, but they are used to provide network security solution in use case scenarios ranging from smart home to smart cities.

In [101], authors provide a secure virtual framework that was built in a smart home environment. The proposed framework is created to be further applied on all virtual smart use cases, from smart cars to smart factories. Their research projects data in a similar manner to our work: use Pis equipped with environmental sensors to collect direct readings, such as temperature, pressure, and upload to cloud server via a high-level protocol. The communications occur using a mixture of protocols: SSH combined with HTTP, which essentially forms HTTPS.

In a smart home use case, smart fridge and smart thermostats, such as Nest, only needs to collect temperature reading and upload the reading to the cloud server. In a smart lab scenario, real-time temperature and pressure readings are constantly uploaded to cloud server. Researchers and lab administrators rely on these readings to preserve lab environments. So even we used Pis, the usage of such a specific device can be generalized. The behavior of the Rainbow HAT resembles the characteristics of those smart devices that execute one-dimensional jobs. We collected our data in both smart home and smart lab environments. Since most active smart devices network behavior can be dissected using NetFlow, which is designed by Cisco, we monitor the NetFlows of these devices and inject real cyberattacks. We are applying a feature engineering solution in NFStream, which is a flow-based feature generation tool.

**Figure 3:**
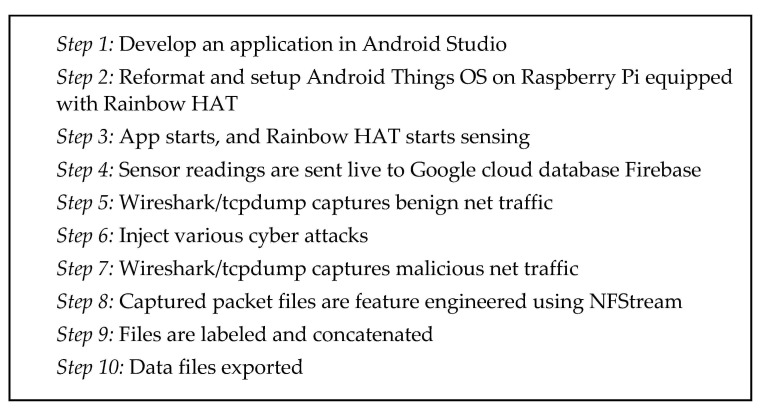
Data collection process.

**Figure 4:**
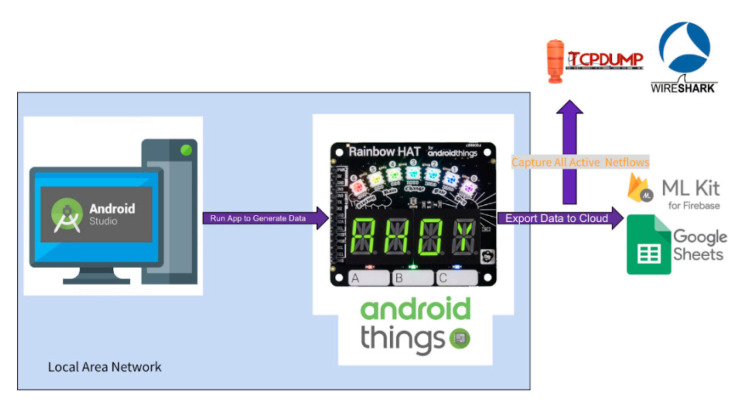
Flow of data in the collection process.

**Figure 5:**
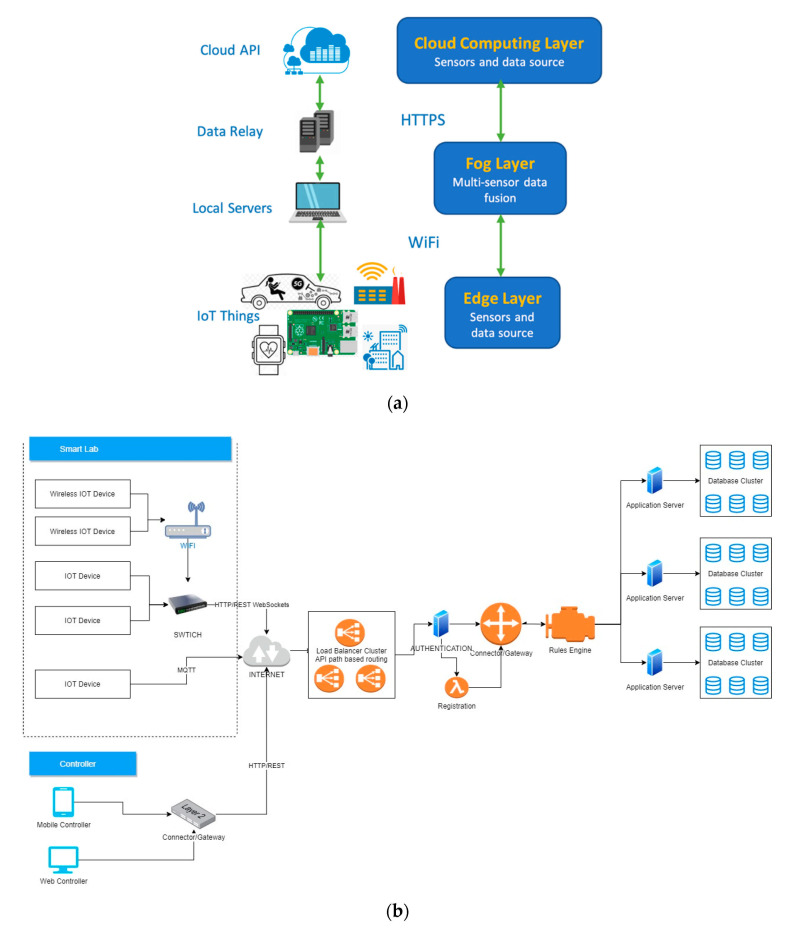
(**a**) The network architecture of CCD-INID-V1. (**b**) Flow of data in a typical IoT network architecture.

As listed in Figure 3, we developed our application on an Android Studio, which is the official integrated development environment (IDE) for the Google-owned Android operating system [102]. We require the application to initiate smart sensors to capture environmental data, and transmit to a cloud-based database, as shown in Figure 4 and Figure 5. The smart sensors originate from a smart-board, Rainbow HAT [103], which is equipped directly on the mini-computer, Raspberry Pi version 3B [104], running on the open-sourced Android Things operating system [105]. Every 2 s, the sensor board captures moisture and temperature of the surroundings. A webserver installed with Wireshark is used to listen to the network traffic in and out of the smart devices. The devices are connected to the webserver through Android Debug Bridge (adb). At random time intervals and using multiple source devices, which include both physical and virtual bots, we launched multiple cyberattacks at the target device. Further details about the attacks are described in Section 3.2.2. We used 4 Raspberry Pis and collected data in two smart environments: smart home and smart lab. All web traffic in and out from the smart devices is exchanged over WiFi connections. The raw captured data totals over 50 GB. The raw data is then converted, and feature engineered using an open-source library, NFStream [106], which is described in detail in Section 3.2.3. After feature engineering, we are able to get 83 features. After labeling and concatenation, we produce the final data file for further experiments.

Sensor readings are encrypted and transmitted through an authenticated channel with random-path-based routing to ensure data privacy. We established handshake and key exchanges using a built-in application programming interface (API) in Android Studio connected to Firebase. We organize data using the rules engine in Firebase to prevent data-injection attacks. The flow of data can be seen in Figure 4.

Based on our security architecture, as shown in Figure 5a, we are mainly focusing on the transmissions between edge devices with cloud servers, where the analysis computing is conducted. At the edge Layer, which contains live sensors, data is originated from the IoT things. By communicating through WiFi and adb port forwarding, we not only monitor the data, but we manufacture features at the local server, hence computing at the fog layer. In smart homes and smart labs, WiFi is one of the most widely used short-range transmission protocols, which also include RFID, WLAN, 6LowWPAN, ZigBee, Bluetooth, NFC, and Z-Wave [107]. The sensors have a direct channel to communicate via HTTPS with the cloud server, where the database is located. In this sense, we are using a hybrid format of computing at both fog and cloud layers [47]. To show that our method is able to identify patterns from traffic through information-hiding, we chose HTTPS as end-to-end communication protocol over HTTP. We want to see how well our method is able to perform without compromising the privacy of users.

As summarized by [108], long-range (higher level) transmission protocols include MQTT, CoAP, AMQP and HTTP(S). In terms of messages size, MQTT can hold the least amount and HTTP(S) can hold the largest. Since we are proposing a solution that is applicable in any IoT environments, from smart home to smart cities, we considered the various long-range protocols. Given the universal usage of HTTP(S), we selected HTTP(S) as our choice of transmission protocol. HTTP(S) is a part of the IP suite of TCP/IP. As the most widely used transmission protocol in the world, TCP/IP includes HTTP, HTTPS, FTP, and MQTT. HTTPS offers the advantage of transmitting the largest message size along with end-to-end information-hiding. With the advancement of technologies such as 5G, we do not necessarily need to reduce message size. Furthermore, we want to show how we are able to detect anomalies without the need to identify what is inside a packet. In other words, we are able to identify threats while ensuring consumer privacy. Many users use TCP/IP protocols to address problems that are found in IoT use cases [109,110,111,112,113,114].

In [109], Alavi et al. apply MQTT along with TCP/IP to transmit data in their data collection process. In [110], the author uses WiFi and ZigBee to transmit data between devices within LAN and uses TCP/IP protocols to transmit data between multiple data relays across the internet. Moreover, a lot of smart devices rely on Application Programming Interface (API) services, notably Representational State Transfer (REST) API, to communicate [111,112,113,114]. REST API is mainly implemented on these protocols: HTTP(S), URI, JSON, and XML.

Although we are applying our current method in smart home and smart labs, but our goal is to extend our method and apply to smart campus, smart cities, smart factory, and smart grid/infrastructures.

**Figure 6:**
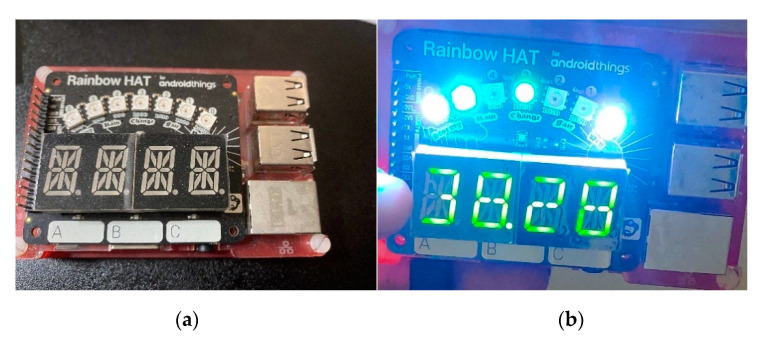
Photo of Raspberry Pi and Rainbow HAT. (**a**) Shutdown status; (**b**) app running.

Even though we only used 4 Pis, as seen in Figure 6a, the usage of such specific devices can be generalized. The behavior of the Rainbow HAT, as shown in Figure 6b resembles the characteristics of those smart devices that execute one-dimensional jobs, such as smart lights, smart thermometer, smart doorlocks without cameras.

#### 3.2.2. List of Attacks

We selected five frequently used attacks in the creation of our dataset. The five attacks are Address Resolution Protocol (ARP) Poisoning, ARP Denial-of-Service (DoS), UDP Flood, Hydra Bruteforce with Asterisk protocol, and SlowLoris. Table 2 describes each attack in detail. Here are the reasonings behind the selection of each attack:

- ARP Poisoning—ARP Poisoning generates minimum web traffic. It is extremely challenging for IDS to pick up the signature of this type of attack. We wanted to see how well our IDS can handle this attack signature with limited trace.
- ARP DoS—This attack leaves plenty of “breadcrumbs” for IDS to pick up. We sent 600,000 messages at our only available socket at a one-second interval continuously for 12 h.
- UDP Flood—Similar to the previous attack, however this attack uses a different protocol. We wanted to test how our IDS handle network traffic with different protocols.
- Hydra Bruteforce with Asterisk protocol—This type of attack attempts to gain authentication using commonly used password combinations. Hydra is a well-known attack toolkit. The Asterisk protocol is an interesting choice for our attack selection because it is a protocol that is standard for voice-over-IP, which relates to many users that rely on communication tools such as Zoom, Skype, WeChat, WhatsApp during the COVID-19 pandemic.
- SlowLoris—SlowLoris is a new representation for low-bandwidth Distributed Denial-of-Service attacks [115]. First developed by a hacker named Robert “RSnake” Hansen, this attack can bring down high-bandwidth servers with a single botnet computer, as evidenced in the 2009 Iranian presidential election [116].

**Table 2:** Attacks on CCD-INID-V1 Dataset.

Name of Attack	Type of Attack	Description
ARP Poisoning	Man-in-the-Middle	ARP poisoning occurs when an attacker sends falsified ARP messages over a local area network (LAN) to link an attacker’s MAC address with the IP address of a legitimate computer or server on the network. Once the attacker’s MAC address is linked to an authentic IP address, the attacker can receive any messages directed to the legitimate MAC address. As a result, the attacker can intercept, modify or block communication to the legitimate MAC address [117].
ARP DoS	DoS	In ARP flooding, the affected system sends ARP replies to all systems connected in a network, causing incorrect entries in the ARP cache. The result is that the affected system is unable to resolve IP and MAC addresses because of the wrong entries in the ARP cache. The affected system is unable to connect to any other system in the network [118].
UDP Flood	DoS	A UDP flood is a type of DoS attack in which a large number of User Datagram Protocol (UDP) packets are sent to a targeted server with the aim of overwhelming the device’s ability to process and respond. The firewall protecting the targeted server can also become exhausted due to UDP flooding, resulting in a DoS to legitimate traffic [119].
Hydra Bruteforce with Asterisk	Bruteforce	Hydra is a parallelized network logon cracker built in various operating systems such as Kali Linux, Parrot, and other penetration testing environments. Hydra works by using different approaches to perform brute-force attacks to guess the right username and password combination [120]. Asterisk supports several standard voice-over-IP protocols, including the Session Initiation Protocol (SIP), the Media Gateway Control Protocol (MGCP), and H. 323. Asterisk supports most SIP telephones, acting both as registrar and back-to-back user agent [121].
SlowLoris	Distributed DoS	SlowLoris is a type of DoS attack tool which allows a single machine to take down another machine’s web server with minimal bandwidth and side effects on unrelated services and ports. SlowLoris tries to keep many connections to the target web server open and hold them open as long as possible. It accomplishes this by opening connections to the target web server and sending a partial request. Periodically, it will send subsequent HTTP headers, adding to, but never completing, the request. Affected servers will keep these connections open, filling their maximum concurrent connection pool, eventually denying additional connection attempts from clients [115].

#### 3.2.3. Feature Engineering Using NFStream

For our dataset, we used NFStream to engineer the features. NFStream is an open-source Python API library that provides flexible and quick feature conversion to make live or offline network data more intuitive. The designers have the broader goal of making the library a common network data analytics framework for researchers providing data reproducibility across experiments, hence standardization. NFStream offers the following benefits:

- **Statistical features extraction**: NFStream provides the post-mortem statistical features (e.g., min, mean, stddev and max of packet size and inter arrival time) and early flow features (e.g., sequence of first n packets sizes, inter arrival times and directions).
- **Flexibility**: NFStream is easily extensible. The project is open-sourced and NFPlugins can be used for feature engineering.

NFStream is built upon the concept of flow-based aggregation. Based on the shared commonalities, such as flow key, transport protocol, VLAN identifier, source and destination IP address, the packets are aggregated into flows. From a flow’s entry until its termination, a flow cache is used to keep trace (e.g., active timeout, inactive timeout). If the entry is present in the flow cache, counters and several other metrics are updated periodically. If flows are generated in both directions, the flow cache applies a bidirectional flow definition, which includes adding counters and metrics for both directions.

In the above schema, NFStream overall architecture is depicted and could be summarized as follows:

- **NFStreamer** is a driver process. The driver’s main responsibility involves setting the overall workflow, which is mostly an orchestration of parallel metering processes.
- **Meters** make up the integral parts to the NFStream framework. Meters transform information gathered through flow-aggregation into statistical features until flow is terminated by expiration (active timeout, inactive timeout). After processing (e.g., timestamped, decoded, truncated), raw packets are dispatched across meters.

After processed by Meters, a flow becomes NFlow, the lexicon used in NFStream. New flow features are engineered based on the configurations set by NFStreamer. In Table 3, we list features that are extracted.

The dataset contains 83 features, including source and destination string representation of IP and MAC addresses, flow bidirectional packets accumulator, and multiple timestamps.

### 3.3. Detection_of_IoT_botnet_attacks_N_BaIoT Dataset

#### Dataset Summary

This publicly available dataset is created by the researchers in [48]. The researchers gathered the data from 9 commercial IoT devices infected by Mirai and BASHLITE. The dataset contains 7,062,606 instances and 115 features. However, these features were extracted using an autoencoder extraction tool, Kitsune [122]. The base features before feature extraction are listed in Table 4.

The dataset contains 10 attacks. The first five attacks fall under the parent category of BASHLITE:

(1) BL_Scan: Scanning the network for vulnerable devices
(2) BL_Junk: Sending spam data
(3) BL_UDP: UDP flooding
(4) BL_TCP: TCP flooding
(5) BL_COMBO: Sending spam data and opening a connection to a specified IP address and port

The remaining five attacks are variations of Mirai:

(1) Mirai_Scan: Automatic scanning for vulnerable devices
(2) Mirai_Ack: Ack flooding
(3) Mirai_Syn: Syn flooding
(4) Mirai_UDP: UDP flooding
(5) Mirai_UDPplain: UDP flooding with fewer options, optimized for higher packet-per-second (PPS).

### 3.4. CIRA-CIC-DoHBrw-2020 Dataset

#### Dataset Summary

The dataset has two layers. The traffic is segregated using a feature engineer tool called DoHMeter. DoHMeter classifies traffic as DoH and non-DoH and generates statistical features in the first layer. In the second layer, DoHMeter classifies traffic as either benign or malicious based on time-series. The network traffic are collected in the formats of HTTPS and DoH. To generate traffic, a variety of 10,000 Alexa websites were accessed. Tools such as DNS tunneling and browsers (e.g., Google Chrome, Mozilla Firefox) were used to generate benign data. Tools such as dns2tcp, DNSCat2, and Iodine, which make up the attack classes, were used to generate malicious data.

The features for this dataset are listed in Table 5. The dataset contains 34 features, of which 28 are statistically extracted.

## 4. Experimental Setup

The experiments were executed on a computer platform with specifications including an Intel Xeon W-2195 2.30 GHz 36 cores processor, 251.4 GB of RAM, Quadro RTX8000 with disk space of 2.0 TB, and an operating system Ubuntu 18.04. The DL structure was developed using the Python programming language and utilizing the TensorFlow-GPU library with Keras neural network library. To balance the dataset for better performance, the imbalanced-learn package [123], an open-sourced Python package, was used. To verify the capabilities of the proposed models, we used three datasets: CCD-INID-V1, BaIoT [48], and DoH20 [49].

### 4.1. Data Preparation and Pre-Processing

The data pre-processing begins with selecting a dataset and converting categorial values into numerical data. To avoid data scrutiny, specific feature columns are dropped because of substantial missing values. Since all three datasets have an imbalanced proportion of data between each attack, we applied imbalanced-learn to balance the data. Data is then split into training and test sets using an 80–20 ratio. 80% of data is used for training and the rest is used for testing.

**Figure 7:**
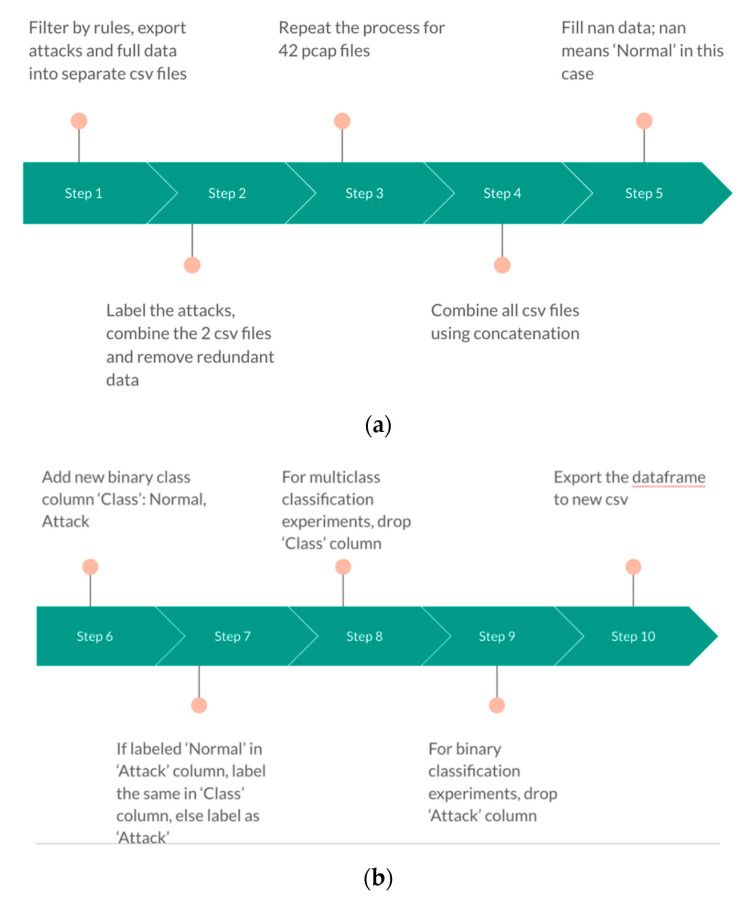
Data preparation for CCD IoT Network Intrusion Dataset V1. (**a**) Steps 1–5; (**b**) Steps 6–10.

The data preparation steps for the CCD-INID-V1 dataset are illustrated in Figure 7. After capturing pcap files with Wireshark, for Step 1 we export the data into separate csv files and add an extra column named ‘Attack’ to specify the nature of the file. Each pcap file can be exported into csv format of which each line represents a packet. Since we captured more than 50 GB of raw data, to avoid the workstation freezing up from heavy workload from Wireshark captures, we applied automatic separation of files with a ceiling of 2 GB of file size. From Step 2 onwards, we proceed with the process using a Jupyter Notebook file with the assistance of the Pandas library. In Step 2, we combine attack labeled csv files with csv files that carry benign traffic. We repeat this process for the 42 csv files in Step 3. In the next step, we combine all the attacks with all the benign traffic by applying concatenation. In Step 5, since we labeled all attacks, any missing value in the ‘Attack’ column is benign traffic. Therefore, we load in the files as dataframes and label them as ‘Normal.’ From Step 6 to Step 9, the procedures vary depending on whether we export an anomaly dataset for binary classification or an attack-based dataset for multiclass classification. Starting with Step 6, since in an anomaly dataset, the traffic is essentially grouped into either as ‘Normal’ traffic or an ‘Attack,’ therefore if we spot ‘Normal’ labels from the ‘Attack’ column, we continue to apply ‘Normal’ labeling in the new column ‘Class.’ Otherwise, we label the packet as ‘Attack.’ If we are exporting a multiclass dataset, we execute Step 8. If we export a binary dataset, we proceed with Step 9. Eventually, we export the output file and conclude the data preparation procedure.

For pre-processing the CCD-INID-V1 dataset, the steps are quick. Since no missing values are incurred from any feature columns, we need to convert the data into numerical values. The target column is classified as either ‘0’ or ‘1’ for anomaly detection or a range from ‘0’ to ‘5’ for multiclass attack-based detection.

In the process of preparing the Balot dataset, we encountered a problem. Since the dataset contains traffic from 9 different devices and half of the attacks were missing for several devices. To ensure we could experiment on as many attacks as possible, we chose the data from Danmini Doorbell, which carries all 10 attack types. However, since each attack is separated by folders and benign traffic is a generic csv file for all of the devices, we had to combine the attack files with the benign traffic using Pandas as well. Since the dataset is originated from 12 base features, listed in Table 3, and converted into 115 features with the help of an autoencoder, there are no missing values in the dataset and we only needed to drop the first sequential column before wrapping up the preparation process. For pre-processing, we converted any non-numeric values into categorical values before converting them to numeric values. We applied this dictionary pairing for the multiclass labeling: {‘Normal’: 10, ‘BL_combo’: 0, ‘BL_junk’: 1, ‘BL_scan’: 2, ‘BL_tcp’: 3, BL_udp: 4, ‘Miral_ack’: 5, ‘Mirai_scan’: 6, ‘Mirai_syn’: 7, ‘Mirai_udp’: 8, ‘Mirai_ack’: 9}.

For the DoH20 Dataset, we apply different procedures for the anomaly dataset and the multiclass dataset. The DoH20 dataset contains 4 main files for binary classification: l1doh, l1nondoh, l2benign and l2malicious. The research group that created this dataset also produced a feature engineering toolkit named DoHMeter, which produces 28 features on any pcap file. The second file contains data before applying the toolkit whereas the first file is the end result after application. The files ‘l2benign’ and ‘l2malicious’ contain the features generated from the toolkit as well. We only needed to combine the malicious and benign files before training and testing. However, we had to drop the feature columns of ‘Standard Deviation of Request/response time difference’ and ‘Standard Deviation of Request/response time difference’ due to missing values. For the multiclass dataset, there were three malicious files given, named ‘dns2tcp,’ ‘DNSCat2,’ and ‘Iodine.’ All three names specify the tools used for attacks. The attacks were carried out on 4 servers: AdGuard, Cloudfare, GoogleDNS and Quad9. We treat each of these tools as a type of attack. The three attacks are combined with benign traffic into a group of 4 classes.

### 4.2. Metrics Used for Evaluations

In this research, two types of classifications were conducted: binary and multi-class. Normal and anomaly are the two classes in binary classification. For the CCD-INID-V1 dataset, the classes include the 5 attacks and the normal traffic. A total of 11 classes are available for multiclass classification for the Balot dataset. The DoH20 dataset contains 4 class: 3 attacks and 1 normal.

We apply the confusion matrix to analyze the performance ontology, which is based on truly or falsely classified values. If a value is classified as true positive (TP), it means the attack packets has been correctly detected. If a benign packet has been falsely classified, then the packet is labeled as false positive (FP). Packet classified as true negative (TN) means that benign traffic has been recognized as normal by the detector. If a value is categorized as false negative (FN), it means the attack has not been spotted by the detector and the value is classified as benign traffic. If all values fall into TP and TN categories, then the IDS reaches the most optimal state. However, if an IDS has substantial FP and FN, then we would rather have more FP than FN.

For performance testing, we use metrics such as accuracy, detection rate, precision, recall, F1-score, and AUC. But we also consider the CPU/GPU memory consumed, training and testing losses over epochs, and computation runtimes.

## 5. Results

In this section, we compare the performances of our models with the traditional ML algorithms when applied on the three datasets. We refer to the three datasets as CCD-INID-V1, Balot, and DoH20, respectively.

### 5.1. Feature Importance

**Figure 8:**
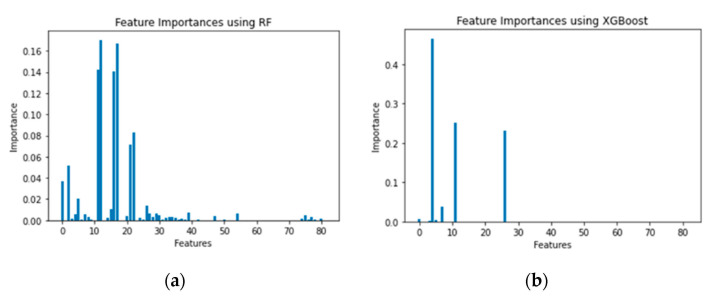
Feature importance on CCD-INID-V1 dataset. (**a**) RF and (**b**) XGBoost.

**Figure 9:**
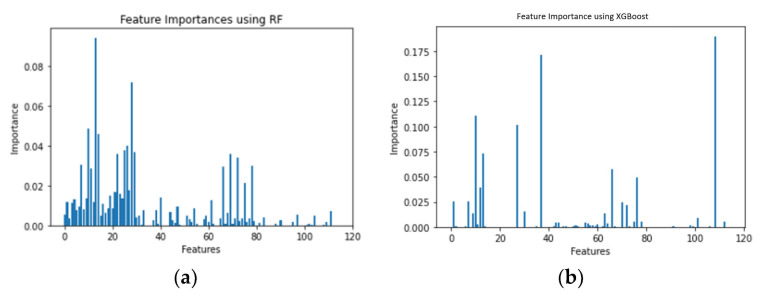
Feature importance on Balot dataset. (**a**) RF and (**b**) XGBoost.

**Figure 10:**
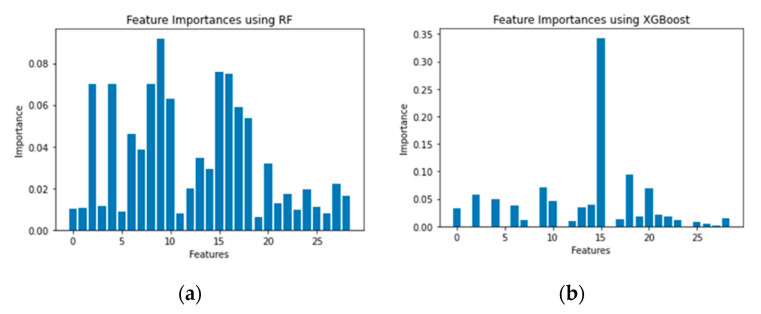
Feature importance on DoH20 dataset. (**a**) RF and (**b**) XGBoost es.

Figure 8, Figure 9 and Figure 10 show the feature importance using the RF and XGboost on three datasets. After dimensionality reduction, we were able to reduce feature size to 41 when using RF and to 7 when using XGBoost without compromising the detection accuracies on CCD-INID-V1 dataset. As for the Balot dataset, we reduced feature size from 115 to 102 using RF. We reduced feature size to just 24 using XGBoost. On DoH20 dataset, we reduced feature size from 29 to 15 using RF whereas with XGBoost, we reduced feature size to just 11.

### 5.2. Training, Testing Loss and Accuracy over Epochs

As we see in Table 6, for 10 epochs of training and testing, RCNN was able to achieve the highest predicting accuracy of 0.9563 with the lowest loss of 0.7005 on CCD-INID-V1 in the 5th epoch. Prediction accuracy of 0.9996 is reached in 6th epoch with a low testing loss of 0.0064 when experimenting on the Balot dataset. On the DoH20 dataset, RCNN achieved a testing accuracy of 0.9818 in epoch 3 even though it only gained a training accuracy of 0.7117 in the same epoch. RCNN reached the best training and testing accuracy in epoch 4 while keeping the losses low.

As we can see from Table 7, the training and testing accuracies of XCNN are identical to that of RCNN. However, when taking the feature reduction into consideration, XCNN was able to achieve this with reduced features on all three datasets.

### 5.3. Confusion Matrix Comparisons

Table 8 and Table 9 show the confusion matrices for binary classification. Table 10, Table 11 and Table 12 carry the confusion matrices for the multiclass classification. For the binary classifications, ‘0’ stands for normal traffic and ‘1’ stands for an anomaly. As for the multiclass classifications, ‘0’ denotes the normal traffic while other integer labels denote various types of attacks.

As we find from Table 8, RCNN and XCNN performed quite well on all three datasets. We obtained reasonably low FP and FN. XCNN performed better than RCNN on the CCD-INID-V1 and DoH20 datasets while RCNN outperforms XCNN slightly on the Balot dataset.

For binary classifications, we compared the results with 4 traditional ML algorithms: KNN, NB, LR and SVM. From Table 9, we can identify that KNN consistently performed well across the three datasets. NB achieved same detection rate as KNN on the DoH20 dataset but struggled with CCD-INID-V1 and Balot. For both CCD-INID-V1 and Balot dataset, NB were unable to detect many attack packets and raised a lot of false alarms. LR didn’t perform well for all datasets as compared with the other algorithms with the exception of SVM. SVM achieved the worst results on all the datasets. Looking at the confusion matrixes, RCNN and XCNN detected more anomalies and raised lesser false alarms than the other generic algorithms over the three datasets; except for the fact that KNN performed better on the CCD-INID-V1 dataset.

For multiclass classifications, we compared the results with 3 traditional ML algorithms: KNN, NB, and LR. In Table 10, we can see that RCNN and XCNN did not do as well as KNN, NB, and LR. In Table 11 and Table 12, the same pattern is found as Table 10.

### 5.4. Comparison of Precision, Recall, F1-Score

Table 13 shows the performance of RCNN and XCNN for binary classification. Table 14 shows results from multiclass classifications.

From Table 13, we find that RCNN and XCNN achieved high precision, recall and F1-scores than the other traditional algorithms on the three datasets except for KNN on CCD-INID-V1. However, when we consider the total computation time, which includes training time and prediction time, we discover that RCNN and XCNN used low timespan to gain high scores. For CCD-INID-V1, LR, NB and SVM trained extremely quick but were unable to get high scores. KNN achieved high scores at the cost of high prediction time. For Balot, SVM and LR took almost 20 min to train, but could not beat the scores of RCNN, XCNN and KNN. Even though KNN got high scores, the training time was five times more to that of RCNN and XCNN and prediction time was 1000% to that of RCNN and XCNN. NB took less time to train and predict than the other generic algorithms but failed to gain high scores. Notably, for the DoH20 dataset, SVM does a good job catching the malicious packets but fail to do so for the normal packets.

For multiclass classification, as shown in Table 14, RCNN and XCNN fail to outperform the traditional ML algorithms. Although KNN was able to get the highest scores, the tradeoff is high computational power and runtime. For instance, for the Balot dataset, KNN used 1081 min to achieve similar results as LR, which took only 150 s, and that of NB, which only took 1.43 s.

### 5.5. Comparison of ROC and AUC

AUC is the entire two-dimensional area under the ROC. ROC is a curve that measures two parameters, the true positive rate and the false positive rate, to show how a classification model performs. The x-axis depicts the false positive rate, and the y-axis depicts the true positive rate. AUC ranges from 0 to 1. If the AUC has a value of 0.0, it means the model makes 100% incorrect predictions; whereas a value of 1.0 means the model has perfect predictions. A value of 0.5 means the model makes no separation of classes. AUC is a desirable form of measure because AUC offers a scaled comparison instead of absolute values and AUC measures the model’s predictive outcomes without taking account of classification thresholds.

**Figure 11:**
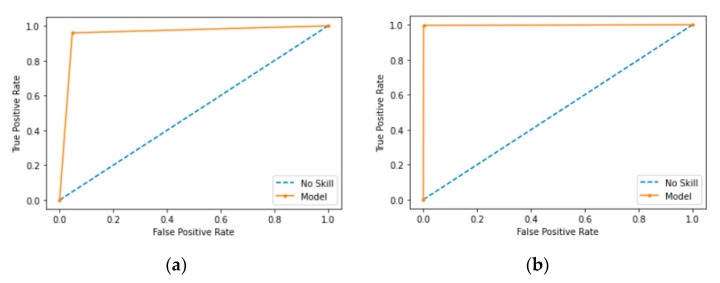
ROC diagrams for results of RCNN and XCNN on CCD-INID-V1 dataset. (**a**) ROC of RCNN; (**b**) ROC of XCNN.

ROC is highlighted in orange. Figure 11 shows ROC curves for XCNN and RCNN when applied on the CCD-INID-V1 dataset. As shown, the proposed models show a reasonable performance in Figure 11a but near perfection in Figure 11b, with AUC close to the value of 1.0.

**Figure 12:**
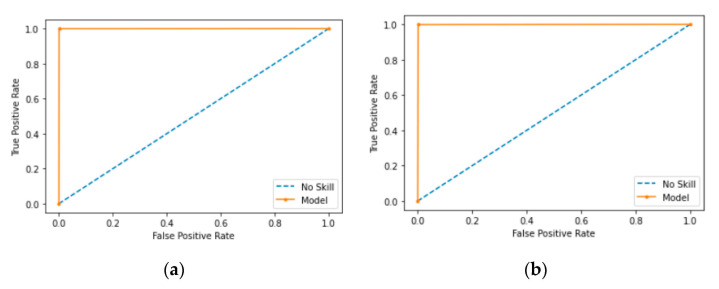
ROC diagrams for results of RCNN and XCNN on Balot dataset. (**a**) ROC of RCNN; (**b**) ROC of XCNN.

**Figure 13:**
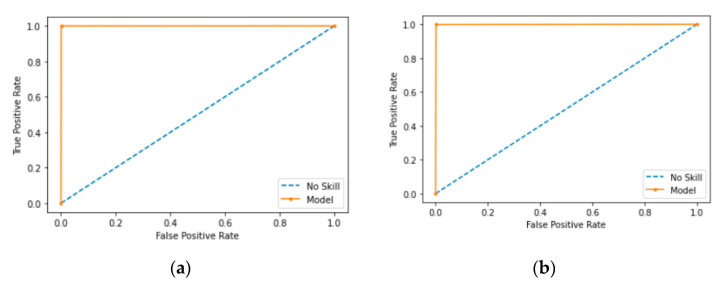
ROC diagrams for results of RCNN and XCNN on DoH20 dataset. (**a**) ROC of RCNN; (**b**) ROC of XCNN.

Figure 12 and Figure 13 show ROC curves on the Balot dataset and the DoH20 dataset, respectfully. As shown, the AUC shows a near perfect results in both cases.

### 5.6. Efficiency Comparisons

Table 15 contains information extracted from Table 13. This table shows the total runtimes of the three models that have the highest precision, recall, F1-score consistently throughout the anomaly detection experiments.

We compare the computational time taken by RCNN and XCNN when training and detecting anomalies on the three datasets. We find that RCNN consistently takes lesser time than XCNN, but considering the size of the datasets, the computation time of XCNN does not increase proportionally as RCNN does.

When compared with KNN, we find that RCNN and XCNN were much more efficient. For the CCD-INID-V1 dataset, XCNN’s computational time was only 10.8% to that of KNN, and RCNN’s computation time was only 6.82% to that of KNN. For the Balot dataset, XCNN’s computation time was only 0.702% to that of KNN, and RCNN’s was just 0.696% to that of KNN. For the DoH20 dataset, XCNN used 1.52% amount of time of what KNN used while RCNN only used 0.74% amount of time to KNN. On average, that’s a 91.74% of reduction to computational resources for RCNN from KNN for all three datasets and a 86.98% reduction for XCNN from KNN.

From the experimental results we find that RCNN and XCNN perform extremely well when applied on anomaly detection but fail to get accurate predictions for attack-based detections. Even though more diverse approaches must be examined, we are able to show that our method is able to significantly reduce computational time by reducing significant features and maintain high detection rate with minimum false alarms when dealing with anomaly detection. This is significant especially when dealing with zero-day attacks, when the signature of new malicious traffic is unrecognizable.

## 6. Conclusions

In this research, we created a dataset using IoT networks with real smart sensors. The dataset mimics the real-world network behavior and attacks. We propose a DL-based hybrid lightweight model for anomaly detection and multi-attack classification. We combine two popular embedded feature selection methods, the RF and XGBoost, with the CNN to form the hybrid model. The model is used to elicit the most important features. A comparative analysis of performances is given when we apply our model with other traditional ML algorithms on three IoT-network-based datasets. While the proposed models fail to outperform the traditional ML algorithms for multi-attack classification, they outperform the traditional methods for cyber anomaly detection on all three IoT datasets.

We achieved AUC scores of 0.956 with a runtime of 32.28 s on CCD-INID-V1, 0.999 with a runtime of 71.46 s on Balot, and 0.986 with a runtime of 35.45 s on DoH20 using RCNN. We obtained AUC scores of 0.998 with runtime of 51.38 s on CCD-INID-V1, 0.999 with runtime of 72.12 s on Balot, and 0.999 with runtime of 72.91 s on DoH20 using XCNN. Compared to KNN, XCNN required 86.98% less computational time and RCNN required 91.74% less computational time to achieve equal or better accurate anomaly detections. Notably when experimenting on the Balot dataset, even though KNN got high scores, the training time was five times more to that of RCNN and XCNN and prediction time was 1000% to that of RCNN and XCNN. The low train time and low predict time is crucial for the deployment of our IDS. Our IDS can be placed at central server as well as resource-constrained edge devices. Our lightweight IDS require low retrain time and hence decreases reaction time to new attacks.

In the future, we plan to explore other avenues to reduce, select or extract features to achieve better attack-based detection for our IDS. In our first version of the dataset, we monitor the network behavior of IoT things in smart home and smart lab. The devices perform straightforward tasks to generate telemetry. However, we did not include multi-faucet data such as a homeowner surfing internet on smart phone or a lab researcher gathering resource through browsers. In an effort to make our dataset more complete and more realistic, we plan to include more user behaviors and more use case scenarios in our next version.

## Figures and Tables

**Table 1:** A comparison of related work.

Approach	Dataset	Dimension Reduction	Anomaly/Multiclass	Lightweight	IDS	IoT IDS
LASSO [94]	AWID [95]	Yes	N/A	Yes	Yes	No
Auto-encoder [86]	Image-based datasets	Yes	Multiclass	No	No	No
Auto-encoder [87]	Image-based datasets	Yes	Multiclass	No	No	No
Auto-encoder [88]	N/A	Yes	Anomaly	No	No	No
JG NSGA-II, CNN+LSTM [89]	CISIDS2017 [85]	Yes	Anomaly	No	Yes	Yes
GRU, Text-CNN [90]	KDD99 [17] and the ADFA-LD [91]	No	Both	Yes	Yes	Yes
Hybrid, LSTM [92]	CICDDoS2019 [93]	No	Anomaly	No	Yes	No
Our proposed work	CCD-INID-V1, Balot [48], DoH20 [49]	Yes	Both	Yes	Yes	Yes

**Table 3:** Features generated for CCD-INID-V1 dataset [106].

Features	Data Type	Description
id	data	Flow identifier
expiration_id	data	Identifier of flow expiration trigger. Can be 0 for idle_timeout, 1 for active_timeout or −1 for custom expiration.
Src_ip	str	Source IP address string representation.
Src_mac	str	Source MAC address string representation.
Src_oui	str	Source Organizationally Unique Identifier string representation.
Src_port	int	Transport layer source port.
Dst_ip	str	Destination IP address string representation.
Dst_mac	str	Destination MAC address string representation.
Dst_oui	str	Destination Organizationally Unique Identifier string representation.
Dst_port	int	Transport layer destination port.
Protocol	int	Transport layer protocol.
Ip_version	int	IP version.
Vlan_id	int	Virtual LAN identifier.
Bidirectional_first_seen_ms	int	Timestamp in milliseconds on first flow bidirectional packet.
Bidirectional_last_seen_ms	int	Timestamp in milliseconds on last flow bidirectional packet.
Bidirectional_duration_ms	int	Flow bidirectional duration in milliseconds.
Bidirectional_packets	int	Flow bidirectional packets accumulator.
Bidirectional_bytes	int	Flow bidirectional bytes accumulator (depends on accounting_mode).
Src2dst_first_seen_ms	int	Timestamp in milliseconds on first flow src2dst packet.
Src2dst_last_seen_ms	int	Timestamp in milliseconds on last flow src2dst packet.
Src2dst_duration_ms	int	Flow src2dst duration in milliseconds.
Src2dst_packets	int	Flow src2dst packets accumulator.
Src2dst_bytes	int	Flow src2dst bytes accumulator (depends on accounting_mode).
Dst2src_first_seen_ms	int	Timestamp in milliseconds on first flow dst2src packet.
Dst2src_last_seen_ms	int	Timestamp in milliseconds on last flow dst2src packet.
Dst2src_duration_ms	int	Flow dst2src duration in milliseconds.
Dst2src_packets	int	Flow dst2src packets accumulator.
Dst2src_bytes	int	Flow dst2src bytes accumulator (depends on accounting_mode).
Application_name	str	nDPI detected application name.
application_category_name	str	nDPI detected application category name.
application_is_guessed	int	Indicates if detection result is based on pure dissection or on a port-based guess.
Requested_server_name	str	Requested server name (SSL/TLS, DNS, HTTP)
client_fingerprint	str	Client fingerprint (DHCP fingerprint for DHCP, JA3 for SSL/TLS and HASSH for SSH).
Server_fingerprint	str	Extracted user agent for HTTP or User Agent Identifier for QUIC
content_type	str	Extracted HTTP content type
bidirectional_min_ps	int	Flow bidirectional minimum packet size (depends on accounting_mode).
Bidirectional_mean_ps	float	Flow bidirectional mean packet size (depends on accounting_mode).
Bidirectional_stdev_ps	float	Flow bidirectional packet size sample standard deviation (depends on accounting_mode).
Bidirectional_max_ps	int	Flow bidirectional maximum packet size (depends on accounting_mode).
Src2dst_min_ps	int	Flow src2dst minimum packet size (depends on accounting_mode).
Src2dst_mean_ps	float	Flow src2dst mean packet size (depends on accounting_mode).
Src2dst_stdev_ps	float	Flow src2dst packet size sample standard deviation (depends on accounting_mode).
Src2dst_max_ps	int	Flow src2dst maximum packet size (depends on accounting_mode).
Dst2src_min_ps	int	Flow dst2src minimum packet size (depends on accounting_mode).
Dst2src_mean_ps	float	Flow dst2src mean packet size (depends on accounting_mode).
Dst2src_stdev_ps	float	Flow dst2src packet size sample standard deviation (depends on accounting_mode).
Dst2src_max_ps	int	Flow dst2src maximum packet size (depends on accounting_mode).
Bidirectional_min_piat_ms	int	Flow bidirectional minimum packet inter arrival time.
Bidirectional_mean_piat_ms	float	Flow bidirectional mean packet inter arrival time.
Bidirectional_stdev_piat_ms	float	Flow bidirectional packet inter arrival time sample standard deviation.
Bidirectional_max_piat_ms	int	Flow bidirectional maximum packet inter arrival time.
Src2dst_min_piat_ms	int	Flow src2dst minimum packet inter arrival time.
Src2dst_mean_piat_ms	float	Flow src2dst mean packet inter arrival time.
Src2dst_stdev_piat_ms	float	Flow src2dst packet inter arrival time sample standard deviation.
Src2dst_max_piat_ms	int	Flow src2dst maximum packet inter arrival time.
Dst2src_min_piat_ms	int	Flow dst2src minimum packet inter arrival time.
Dst2src_mean_piat_ms	float	Flow dst2src mean packet inter arrival time.
Dst2src_stdev_piat_ms	float	Flow dst2src packet inter arrival time sample standard deviation.
Dst2src_max_piat_ms	int	Flow dst2src maximum packet inter arrival time.
Bidirectional_syn_packets	int	Flow bidirectional syn packet accumulators.
Bidirectional_cwr_packets	int	Flow bidirectional cwr packet accumulators.
Bidirectional_ece_packets	int	Flow bidirectional ece packet accumulators.
Bidirectional_urg_packets	int	Flow bidirectional urg packet accumulators.
Bidirectional_ack_packets	int	Flow bidirectional ack packet accumulators.
Bidirectional_psh_packets	int	Flow bidirectional psh packet accumulators.
Bidirectional_rst_packets	int	Flow bidirectional rst packet accumulators.
Bidirectional_fin_packets	int	Flow bidirectional fin packet accumulators.
Src2dst_syn_packets	int	Flow src2dst syn packet accumulators.
Src2dst_cwr_packets	int	Flow src2dst cwr packet accumulators.
Src2dst_ece_packets	int	Flow src2dst ece packet accumulators.
Src2dst_urg_packets	int	Flow src2dst urg packet accumulators.
Src2dst_ack_packets	int	Flow src2dst ack packet accumulators.
Src2dst_psh_packets	int	Flow src2dst psh packet accumulators.
Src2dst_rst_packets	int	Flow src2dst rst packet accumulators.
Src2dst_fin_packets	int	Flow src2dst fin packet accumulators.
Dst2src_syn_packets	int	Flow dst2src syn packet accumulators.
Dst2src_cwr_packets	int	Flow dst2src cwr packet accumulators.
Dst2src_ece_packets	int	Flow dst2src ece packet accumulators.
Dst2src_urg_packets	int	Flow dst2src urg packet accumulators.
Dst2src_ack_packets	int	Flow dst2src ack packet accumulators.
Dst2src_psh_packets	int	Flow dst2src psh packet accumulators.
Dst2src_rst_packets	int	Flow dst2src rst packet accumulators.
Dst2src_fin_packets	int	Flow dst2src fin packet accumulators.

**Table 4:** Base features of the detection_of_IoT_botnet_attacks_N_BaIoT Dataset [48].

Features	Data Type	Description
H	Stream aggregation	Stats summarizing the recent traffic from this packet’s host (IP)
HH	Stream aggregation	Stats summarizing the recent traffic going from this packet’s host (IP) to the packet’s destination host.
HpHp	Stream aggregation	Stats summarizing the recent traffic going from this packet’s host+port (IP) to the packet’s destination host+port. Example 192.168.4.2:1242 → 192.168.4.12:80
HH_jit	Stream aggregation	Stats summarizing the jitter of the traffic going from this packet’s host (IP) to the packet’s destination host.
L5, L3, L1, …	Time-frame	The decay factor Lambda used in the damped window
Weight	Statistics	The weight of the stream (can be viewed as the number of items observed in recent history)
Mean	Statistics	The weight of the stream (can be viewed as the number of items observed in recent history)
Std	Statistics	The weight of the stream (can be viewed as the number of items observed in recent history)
Radius	Statistics	The root squared sum of the two streams’ variances
Magnitude	Statistics	The root squared sum of the two streams’ means
Cov	Statistics	an approximated covariance between two streams
pcc	Statistics	an approximated covariance between two streams

**Table 5:** Features of the CIRA-CIC-DoHBrw-2020 Dataset [49].

Features	Data Type	Description
SourceIP	str	IP of source
DestinationIP	str	IP of destination
SourcePort	str	Source port number
DestinationPort	str	Port number of destination
TimeStamp	str	Systime
Duration	str	Duration of packet in transit
FlowBytesSent	str	Number of flow bytes sent
FlowSentRate	float64	Rate of flow bytes sent
FlowBytesReceived	float64	Number of flow bytes received
FlowReceivedRate	float64	Rate of flow bytes received
PacketLengthVariance	float64	Variance of packet length
PacketLengthStandardDeviation	float64	Standard deviation of packet length
PacketLengthMean	float64	Mean packet length
PacketLengthMedian	float64	Median packet length
PacketLengthMode	float64	Mode packet length
PacketLengthSkewFromMedian	float64	Skew from median packet length
PacketLengthSkewFromMode	float64	Skew from mode packet length
PacketLengthCoefficientofVariation	float64	Coefficient of variation of packet length
PacketTimeVariance	float64	Variance of packet time
PacketTimeStandardDeviation	float64	Standard deviation of packet time
PacketTimeMean	float64	Mean packet time
PacketTimeMedian	float64	Median packet time
PacketTimeMode	float64	Mode packet time
PacketTimeSkewFromMedian	float64	Skew from median packet time
PacketTimeSkewFromMode	float64	Skew from mode packet time
PacketTimeCoefficientofVariation	float64	Coefficient of variation of packet time
ResponseTimeTimeVariance	float64	Variance of request/response time difference
ResponseTimeTimeStandardDeviation	float64	Standard deviation of request/response time difference
ResponseTimeTimeMean	float64	Mean request/response time difference
ResponseTimeTimeMedian	float64	Median request/response time difference
ResponseTimeTimeMode	float64	Mode request/response time difference
ResponseTimeTimeSkewFromMedian	float64	Skew from median request/response time difference
ResponseTimeTimeSkewFromMode	float64	Skew from mode request/response time difference
ResponseTimeTimeCoefficientofVariation	float64	Coefficient of variation of request/response time difference

**Table 6:** Training, testing loss and accuracy over epochs using RCNN for binary classification.

Datasets	Epochs	Training Accuracy	Training Loss	Testing Accuracy	Testing Loss
CCD-INID-V1	1	0.8883	1.3042	0.9380	0.9850
	2	0.9428	0.9088	0.9500	0.7976
	3	0.9389	0.9761	0.9505	0.7937
	4	0.9376	0.9980	0.9492	0.8128
	5	0.9378	0.9959	0.9563	0.7005
	6	0.9410	0.9443	0.9514	0.7790
	7	0.9452	0.8758	0.9484	0.8259
	8	0.9435	0.9046	0.9504	0.7951
	9	0.9446	0.8881	0.9515	0.7772
	10	0.9456	0.8713	0.9515	0.7773
Balot	1	0.9748	0.0927	0.9981	0.0257
	2	0.9980	0.0202	0.9986	0.0207
	3	0.9985	0.0182	0.9992	0.0114
	4	0.9980	0.0266	0.9986	0.0139
	5	0.9989	0.0153	0.9994	0.0104
	6	0.9989	0.0158	0.9996	0.0064
	7	0.9994	0.0088	0.9990	0.0149
	8	0.9992	0.0125	0.9990	0.0125
	9	0.9993	0.0102	0.9990	0.0165
	10	0.9994	0.0097	0.9989	0.0176
DoH20	1	0.8684	0.5958	0.5002	5.6470
	2	0.5001	7.9952	0.5000	8.0151
	3	0.7117	4.5212	0.9818	0.1519
	4	0.9766	0.1375	0.9863	0.0601
	5	0.5709	6.8518	0.5000	8.0151
	6	0.4998	8.0176	0.5000	8.0151
	7	0.4999	8.0165	0.5000	8.0151
	8	0.5000	8.0158	0.5000	8.0151
	9	0.5002	8.0122	0.5000	8.0151
	10	0.4999	8.0159	0.5000	8.0151

**Table 7:** Training, testing loss and accuracy over epochs using XCNN for binary classification.

Datasets	Epochs	Training Accuracy	Training Loss	Testing Accuracy	Testing Loss
CCD-INID-V1	1	0.8883	1.3042	0.9380	0.9850
	2	0.9428	0.9088	0.9500	0.7976
	3	0.9389	0.9761	0.9505	0.7937
	4	0.9376	0.9980	0.9492	0.8128
	5	0.9378	0.9959	0.9563	0.7005
	6	0.9410	0.9443	0.9514	0.7790
	7	0.9452	0.8758	0.9484	0.8259
	8	0.9435	0.9046	0.9504	0.7951
	9	0.9446	0.8881	0.9515	0.7772
	10	0.9456	0.8713	0.9515	0.7773
Balot	1	0.9748	0.0927	0.9981	0.0257
	2	0.9980	0.0202	0.9986	0.0207
	3	0.9985	0.0182	0.9992	0.0114
	4	0.9980	0.0266	0.9986	0.0139
	5	0.9989	0.0153	0.9994	0.0104
	6	0.9989	0.0158	0.9996	0.0064
	7	0.9994	0.0088	0.9990	0.0149
	8	0.9992	0.0125	0.9990	0.0125
	9	0.9993	0.0102	0.9990	0.0165
	10	0.9994	0.0097	0.9989	0.0176
DoH20	1	0.8684	0.5958	0.5002	5.6470
	2	0.5001	7.9952	0.5000	8.0151
	3	0.7117	4.5212	0.9818	0.1519
	4	0.9766	0.1375	0.9863	0.0601
	5	0.5709	6.8518	0.5000	8.0151
	6	0.4998	8.0176	0.5000	8.0151
	7	0.4999	8.0165	0.5000	8.0151
	8	0.5000	8.0158	0.5000	8.0151
	9	0.5002	8.0122	0.5000	8.0151
	10	0.4999	8.0159	0.5000	8.0151

**Table 8:** Confusion matrices of RCNN and XCNN with binary classification.

Datasets	Predictions		Actual Results		Predictions		Actual Results	
CCD-INID-V1	RCNN		Actual		XCNN		Actual	
			0	1			0	1
	Predicted	0	8558	424	Predicted	0	8977	5
		1	361	8621		1	29	8953
Balot	RCNN		Actual		XCNN		Actual	
			0	1			0	1
	Predicted	0	306,212	0	Predicted	0	306,202	12
		1	0	440,287		1	10	440,275
DoH20	RCNN		Actual		XCNN		Actual	
			0	1			0	1
	Predicted	0	8912	70	Predicted	0	9985	16
		1	177	8805		1	8	9993

**Table 9:** Confusion matrices of generic algorithms with binary classification.

Datasets	Predictions		Actual Results		Predictions		Actual Results		Predictions		Actual Results		Predictions		Actual Results	
CCD-INID-V1	KNN		Actual		NB		Actual		LR		Actual		SVM		Actual	
			0	1			0	1			0	1			0	1
	Predicted	0	11,088	0	Predicted	0	7897	3191	Predicted	0	7897	3191	Predicted	0	7897	3191
		1	0	11,829		1	5374	6455		1	5374	6455		1	5374	6455
Balot	KNN		Actual		NB		Actual		LR		Actual		SVM		Actual	
			0	1			0	1			0	1			0	1
	Predicted	0	303,123	2313	Predicted	0	183,728	145,294	Predicted	0	228,758	76,678	Predicted	0	172,832	132,604
		1	3089	437,974		1	122,484	294,993		1	36,060	405,003		1	32,023	409,040
DoH20	KNN		Actual		NB		Actual		LR		Actual		SVM		Actual	
			0	1			0	1			0	1			0	1
	Predicted	0	4038	808	Predicted	0	4038	808	Predicted	0	3415	1431	Predicted	0	3225	1621
		1	319	62,246		1	319	62,246		1	523	62,042		1	3941	58,624

**Table 10:** Confusion matrices for CCD-INID-V1 dataset with multiclass classification.

Approach		0	1	2	3	4	5
RCNN	0	409	0	575	0	0	0
	1	263	0	721	0	0	0
	2	134	0	850	0	0	0
	3	124	0	860	0	0	0
	4	171	0	813	0	0	0
	5	72	0	912	0	0	0
XCNN	0	978	0	0	5	1	0
	1	839	135	3	2	0	5
	2	956	0	19	2	6	1
	3	146	0	0	838	0	0
	4	963	0	1	1	16	3
	5	883	0	0	0	1	100
KNN	0	2867	0	0	0	0	0
	1	0	2674	0	0	0	0
	2	0	0	1958	0	0	0
	3	0	0	0	11,829	0	0
	4	0	0	0	0	2384	0
	5	0	0	0	0	0	1205
NB	0	2867	0	0	0	0	0
	1	0	2674	0	0	0	0
	2	0	0	1958	0	0	0
	3	0	0	0	11,829	0	0
	4	0	0	0	0	2384	0
	5	0	0	0	0	0	1205
LR	0	2867	0	0	0	0	0
	1	0	2674	0	0	0	0
	2	0	0	1958	0	0	0
	3	0	0	0	11,829	0	0
	4	0	0	0	0	2384	0
	5	0	0	0	0	0	1205

**Table 11:** Confusion matrices for Balot dataset with multiclass classification.

Approach		0	1	2	3	4	5	6	7	8	9	10
RCNN	0	0	0	0	0	0	0	9762	0	0	0	0
	1	0	0	0	0	0	0	11,892	0	0	0	0
	2	0	0	0	0	0	0	5740	0	0	0	0
	3	0	0	0	0	0	0	5864	0	0	0	0
	4	0	0	0	0	0	0	18,436	0	0	0	0
	5	0	0	0	0	0	0	21,404	0	0	0	0
	6	0	0	0	0	0	0	20,460	0	0	0	0
	7	0	0	0	0	0	0	21,640	0	0	0	0
	8	0	0	0	0	0	0	24,461	0	0	0	0
	9	0	0	0	0	0	0	47,605	0	0	0	0
	10	0	0	0	0	0	0	20,439	0	0	0	0
XCNN	0	0	0	0	0	0	0	0	0	0	9762	0
	1	0	0	0	0	0	0	0	0	0	11,892	0
	2	0	0	0	0	0	0	0	0	0	5740	0
	3	0	0	0	0	0	0	0	0	0	5864	0
	4	0	0	0	0	0	0	0	0	0	18,436	0
	5	0	0	0	0	0	0	0	0	0	21,404	0
	6	0	0	0	0	0	0	0	0	0	20,460	0
	7	0	0	0	0	0	0	0	0	0	21,640	0
	8	0	0	0	0	0	0	0	0	0	24,461	0
	9	0	0	0	0	0	0	0	0	0	47,605	0
	10	0	0	0	0	0	0	0	0	0	20,439	0
KNN	0	15,071	40	13	2	0	0	0	0	1	1	6
	1	34	7113	2	5	0	1	0	0	0	0	0
	2	15	6	7419	0	0	0	2	1	0	1	64
	3	15	8	5	22,916	1	0	0	0	0	0	1
	4	6	4	1	1	26,342	0	0	0	0	0	1
	5	1	0	0	0	0	4644	1	222	2350	11,663	10
	6	0	0	18	0	0	0	26,917	0	0	0	18
	7	1	2	5	0	0	918	1	23,125	4508	1760	27
	8	1	0	12	0	0	3402	1	4980	47,988	3291	62
	9	0	0	0	0	0	13,108	1	2693	3285	6428	15
	10	3	5	58	0	0	13	0	8	39	12	12,303
NB	0	15,071	40	13	2	0	0	0	0	1	1	6
	1	34	7113	2	5	0	1	0	0	0	0	0
	2	15	6	7419	0	0	0	2	1	0	1	64
	3	15	8	5	22,916	1	0	0	0	0	0	1
	4	6	4	1	1	26,342	0	0	0	0	0	1
	5	1	0	0	0	0	4644	1	222	2350	11,663	10
	6	0	0	18	0	0	0	26,917	0	0	0	18
	7	1	2	5	0	0	918	1	23,125	4508	1760	27
	8	1	0	12	0	0	3402	1	4980	47,988	3291	62
	9	0	0	0	0	0	13,108	1	2693	3285	6428	15
	10	3	5	58	0	0	13	0	8	39	12	12,303
LR	0	15,071	40	13	2	0	0	0	0	1	1	6
	1	34	7113	2	5	0	1	0	0	0	0	0
	2	15	6	7419	0	0	0	2	1	0	1	64
	3	15	8	5	22,916	1	0	0	0	0	0	1
	4	6	4	1	1	26,342	0	0	0	0	0	1
	5	1	0	0	0	0	4644	1	222	2350	11,663	10
	6	0	0	18	0	0	0	26,917	0	0	0	18
	7	1	2	5	0	0	918	1	23,125	4508	1760	27
	8	1	0	12	0	0	3402	1	4980	47,988	3291	62
	9	0	0	0	0	0	13,108	1	2693	3285	6428	15
	10	3	5	58	0	0	13	0	8	39	12	12,303

**Table 12:** Confusion matrices for DoH20 dataset with multiclass classification.

Approach		0	1	2	3
RCNN	0	0	3942	0	0
	1	0	33,542	0	0
	2	0	7229	0	0
	3	0	9243	0	0
XCNN	0	801	2325	567	249
	1	72	31,865	992	613
	2	70	3161	2317	1681
	3	70	3941	1373	3859
KNN	0	4366	334	75	93
	1	130	40,769	423	594
	2	32	182	8643	135
	3	14	249	253	11,152
NB	0	4366	334	75	93
	1	130	40,769	423	594
	2	32	182	8643	135
	3	14	249	253	11,152
LR	0	4366	334	75	93
	1	130	40,769	423	594
	2	32	182	8643	135
	3	14	249	253	11,152

**Table 13:** Comparisons of binary results for precision, recall, F1-score and runtimes for RCNN, XCNN and generic algorithms.

Dataset/Approach		Precision	Recall	F1-Score	Train Time	Predict Time	Total Runtime
CCD-INID-V1/RCNN	0	0.96	0.95	0.96	28.96 s	3.32 s	32.28 s
	1	0.95	0.96	0.96			
CCD-INID-V1/XCNN	0	0.99	0.99	0.99	42.32 s	9.07 s	51.39 s
	1	0.99	0.99	0.99			
CCD-INID-V1/KNN	0	1.00	1.00	1.00	26.1 ms	7 min 53 s	7 min 53 s
	1	1.00	1.00	1.00			
CCD-INID-V1/LR	0	0.60	0.71	0.65	8.57 s	350 ms	8.92 s
	1	0.67	0.55	0.60			
CCD-INID-V1/NB	0	0.60	0.71	0.65	19.9 ms	18.2 ms	38.1 ms
	1	0.67	0.55	0.60			
CCD-INID-V1/SVM	0	0.60	0.71	0.65	22.3 s	34.9 ms	22.33 s
	1	0.67	0.55	0.60			
Balot/RCNN	0	1.00	1.00	1.00	63.23 s	8.24 s	71.47 s
	1	1.00	1.00	1.00			
Balot/XCNN	0	0.99	0.99	0.99	60.03 s	12.10 s	72.13 s
	1	0.99	0.99	0.99			
Balot/KNN	0	0.99	0.99	0.99	5 min 21 s	165 min 41 s	171 min 2 s
	1	0.99	0.99	0.99			
Balot/LR	0	0.86	0.75	0.80	19 min 3 s	2 min 14 s	21 min 17 s
	1	0.84	0.92	0.88			
Balot/NB	0	0.60	0.71	0.65	4 min 32 s	5 min 21 s	9 min 53 s
	1	0.67	0.55	0.60			
Balot/SVM	0	0.84	0.57	0.68	25 min 6 s	3 min 17 s	28 min 23 s
	1	0.76	0.93	0.83			
DoH20/RCNN	0	0.98	0.99	0.99	24 s	11.45 s	35.45 s
	1	0.99	0.98	0.99			
DoH20/XCNN	0	1.00	1.00	1.00	67.45 s	5.46 s	72.91 s
	1	1.00	1.00	1.00			
DoH20/KNN	0	0.93	0.83	0.88	19 ms	79 min 46 s	79 min 46 s
	1	0.99	0.99	0.99			
DoH20/LR	0	0.87	0.70	0.78	16 min 44 s	226 ms	166 min 46 s
	1	0.98	0.99	0.98			
DoH20/NB	0	0.93	0.83	0.88	109 ms	23.6 ms	132.6 ms
	1	0.99	0.99	0.99			
DoH20/SVM	0	0.45	0.67	0.54	50.2 s	36.3 ms	50.24 s
	1	0.97	0.94	0.95			

**Table 14:** Comparisons of multiclass results for precision, recall, F1-score and runtimes for RCNN, XCNN and generic algorithms.

Dataset/Approach		Precision	Recall	F1-Score	Train Time	Predict Time	Total Runtime
CCD-INID-V1/RCNN	0	0.35	0.42	0.38	18.24 s	4.18 s	22.42 s
	1	0	0	0			
	2	0.18	0.86	0.30			
	3	0	0	0			
	4	0	0	0			
	5	0	0	0			
CCD-INID-V1/XCNN	0	0.21	0.99	0.34	16.31	9.66 s	25.97 s
	1	1.00	0.14	0.24			
	2	0.83	0.02	0.04			
	3	0.99	0.85	0.91			
	4	0.67	0.02	0.03			
	5	0.92	0.10	0.18			
CCD-INID-V1/KNN	0	1.00	1.00	1.00	5 min 41 s	5 min 29 s	10 min 70 s
	1	1.00	1.00	1.00			
	2	1.00	1.00	1.00			
	3	1.00	1.00	1.00			
	4	1.00	1.00	1.00			
	5	1.00	1.00	1.00			
CCD-INID-V1/LR	0	1.00	1.00	1.00	20 ms	1 min 6 s	1 min 6 s
	1	1.00	1.00	1.00			
	2	1.00	1.00	1.00			
	3	1.00	1.00	1.00			
	4	1.00	1.00	1.00			
	5	1.00	1.00	1.00			
CCD-INID-V1/NB	0	1.00	1.00	1.00	45.1 ms	43 ms	88.1 ms
	1	1.00	1.00	1.00			
	2	1.00	1.00	1.00			
	3	1.00	1.00	1.00			
	4	1.00	1.00	1.00			
	5	1.00	1.00	1.00			
Balot/RCNN	0	0.00	0.00	0.00	297.10 s	70.11 s	367.21 s
	1	0.00	0.00	0.00			
	2	0.00	0.00	0.00			
	3	0.00	0.00	0.00			
	4	0.00	0.00	0.00			
	5	0.10	1.00	0.19			
	6	0.00	0.00	0.00			
	7	0.00	0.00	0.00			
	8	0.00	0.00	0.00			
	9	0.00	0.00	0.00			
	10	0.00	0.00	0.00			
Balot/XCNN	0	0.00	0.00	0.00	250.01 s	113.86 s	363.87 s
	1	0.00	0.00	0.00			
	2	0.00	0.00	0.00			
	3	0.00	0.00	0.00			
	4	0.00	0.00	0.00			
	5	0.00	0.00	0.00			
	6	0.00	0.00	0.00			
	7	0.00	0.00	0.00			
	8	0.00	0.00	0.00			
	9	0.23	1.00	0.37			
	10	0.00	0.00	0.00			
Balot/KNN	0	0.99	1.00	1.00	531 min 31 s	539 min 28 s	1080 min 59 s
	1	0.99	0.99	0.99			
	2	0.98	0.99	0.99			
	3	1.00	1.00	1.00			
	4	1.00	1.00	1.00			
	5	0.21	0.25	0.23			
	6	1.00	1.00	1.00			
	7	0.75	0.76	0.75			
	8	0.82	0.80	0.81			
	9	0.28	0.25	0.26			
	10	0.98	0.99	0.99			
Balot/LR	0	0.99	1.00	1.00	22 s	2 min 10 s	2 min 32 s
	1	0.99	0.99	0.99			
	2	0.98	0.99	0.99			
	3	1.00	1.00	1.00			
	4	1.00	1.00	1.00			
	5	0.21	0.25	0.23			
	6	1.00	1.00	1.00			
	7	0.75	0.76	0.75			
	8	0.82	0.80	0.81			
	9	0.28	0.25	0.26			
	10	0.98	0.99	0.99			
Balot/NB	0	0.99	1.00	1.00	1.42 s	1.43 s	2.85 s
	1	0.99	0.99	0.99			
	2	0.98	0.99	0.99			
	3	1.00	1.00	1.00			
	4	1.00	1.00	1.00			
	5	0.21	0.25	0.23			
	6	1.00	1.00	1.00			
	7	0.75	0.76	0.75			
	8	0.82	0.80	0.81			
	9	0.28	0.25	0.26			
	10	0.98	0.99	0.99			
DoH20/RCNN	0	0.00	0.00	0.00	42.37 s	8.52 s	50.89 s
	1	0.62	1.00	0.77			
	2	0.00	0.00	0.00			
	3	0.00	0.00	0.00			
DoH20/XCNN	0	0.79	0.20	0.32	42.21 s	8.48 s	50.69 s
	1	0.77	0.95	0.85			
	2	0.44	0.32	0.37			
	3	0.60	0.42	0.49			
DoH20/KNN	0	0.96	0.89	0.93	79 min 45 s	80 min 30 s	160 min 15 s
	1	0.98	0.97	0.98			
	2	0.92	0.96	0.94			
	3	0.93	0.96	0.94			
DoH20/LR	0	0.96	0.90	0.93	28 s	72 min 25 s	72 min 53 s
	1	0.98	0.97	0.98			
	2	0.92	0.96	0.94			
	3	0.93	0.96	0.94			
DoH20/NB	0	0.96	0.90	0.93	27 ms	57.8 ms	84.8 ms
	1	0.98	0.97	0.98			
	2	0.92	0.96	0.94			
	3	0.93	0.96	0.94			

**Table 15:** Detection time used by RCNN, XCNN, and KNN for anomaly detections, measured in seconds.

Dataset	RCNN	XCNN	KNN	Epochs
CCD-INID-V1	32.28 s	51.38 s	7 min 53 s	10
Balot	71.46 s	72.12 s	171 min 2 s	10
DoH20	35.45 s	72.91 s	79 min 46 s	10

## Data Availability

Data is available with the approval from the Center for Cyber Defense at North Carolina A&T State University. Please contact zliu2@aggies.ncat.edu for further information.
